# Investigation of the Gel Properties and Gelation Mechanism of a Surimi Blend Composed of Skipjack Tuna (*Katsuwonus pelamis*) and Purpleback Flying Squid (*Symplectoteuthis oualaniensis*)

**DOI:** 10.3390/foods14040621

**Published:** 2025-02-13

**Authors:** Jianwei Liao, Haohao Shi, Jiamei Wang, Guanghua Xia, Yongqiang Zhao, Gang Yu, Xuanri Shen

**Affiliations:** 1 Hainan Engineering Research Center of Aquatic Resources Efficient Utilization in South China Sea, Key Laboratory of Food Nutrition and Functional Food of Hainan Province, Key Laboratory of Seafood Processing of Haikou, School of Food Science and Technology, Hainan University, Haikou 570228, China; 20223002070@hainanu.edu.cn (J.L.); shihaohao@hainanu.edu.cn (H.S.); 992918@hainanu.edu.cn (J.W.); 2 Collaborative Innovation Center of Provincial and Ministerial Co-Construction for Marine Food Deep Processing, Dalian Polytechnic University, Dalian 116034, China; 3 Sanya Tropical Fisheries Research Institute, Sanya 572018, China; zhaoyq@scsfri.ac.cn (Y.Z.); gyu0928@163.com (G.Y.); 4 College of Food Science and Technology, Hainan Tropical Ocean University, Sanya 572022, China; 990497@hainanu.edu.cn

**Keywords:** surimi, blend, gel properties, mechanism

## Abstract

The objective of the present study was to investigate the gel properties and gelation mechanism of a surimi blend consisting of *Katsuwonus pelamis* and *Symplectoteuthis oualaniensis*. Superior gel properties, including gel strength, cooking loss and water holding capacity, were observed in mixed surimi. With increasing proportions of *K. pelamis* in the blend, an increase in hardness, gumminess and chewiness emerged, which compromised the resilience and whiteness of the gels. The detection of apparent viscosity revealed the shear-thinning properties of mixed surimi. The results of the molecular force measurements and differential scanning calorimetry demonstrated that heterogeneous myofibrillar proteins interacted into rigid protein aggregates with the help of enhanced hydrophobic interactions, subsequently increasing the values of G’ and G”. According to the FTIR spectrum, as the proportion of *K. pelamis* gradually increased, the protein secondary structure of surimi transitioned from a random coil to a *β*-sheet, facilitating the formation of a more ordered network structure. A marked improvement in the microstructure was observed via SEM. Therefore, the incorporation of surimi can be employed to optimize gel properties.

## 1. Introduction

The cumulative output of global fisheries and aquaculture production reached a high level of 232 million tons in 2022, which was driven primarily by the significant expansion of aquaculture in Asia. China remains the top producer in aquatic animal production, accounting for 36%, according to a yearbook report published by the Food and Agriculture Organization [1]. Owing to its ability to reproduce rapidly and probe resources, reaching 1.5 to 2.05 million tons, the Purpleback flying squid (*Symplectoteuthis oualaniensis*) is a commercially significant cephalopod species in the South China Sea [2,3,4]. Like other cephalopod species, the adequate presence of high-quality proteins and low lipid content in *S. oualaniensis* render it highly promising for food application [5].

Surimi, a protein concentrate primarily composed of myofibrillar proteins, is manufactured through successive washing to eliminate undesirable sarcoplasmic proteins, lipids, enzymes and other impure components from minced fish muscle [6]. Surimi-based products are considered suitable patterns for bulk fishes with untapped potential and commonly contain fish balls, kamaboko and fish sausages. Owing to their excellent nutritive value, satisfactory sensory quality and special flavor, the annual demand for restructured fish products has increased continuously [7]. The market potential of *S. oualaniensis* in surimi production is undoubtedly substantial because of its remarkable nutritional value, the presence of luscious muscle and unique flavor components [8].

Gel strength, as one of the essential properties of gelation, serves as an indicator of the quality of surimi gel. The thermal gelation of squid surimi has been investigated in previous studies, revealing its low gel-forming capacity and suboptimal gel strength. These characteristics can be attributed mainly to the higher level of proteolytic activity and the difference in myofibrillar ultrastructure compared with those of other fish species [9,10,11,12]. Therefore, innovative approaches need to be developed for the use of squid as a raw material in the production of surimi products. The gelation characteristics of surimi blends have been previously investigated, demonstrating their superiority over individual components [13]. Reference [7] studied the cooperative gelation effects observed in surimi mixtures and reported that the gel strength and elastic modulus were greater than those of individual surimi samples alone. The superior gel properties of the blend were found to be dependent on both the type and content of the fish used [14]. Reference [13] reported that the inclusion of croaker muscle in a surimi mixture led to a significant increase in the intensity of the myosin heavy chain (MHC) band, thereby increasing gel strength. Notably, very little was found in the application of squid in mixed surimi.

Skipjack tuna (*Katsuwonus pelamis*), an economically viable marine fish species with low cost, spans tropical, subtropical and temperate oceans, including the South China Sea [15]. *K. pelamis* ranks third in catch tons of finfish, reaching 3.1 million tons, followed by Alaskan pollock (*Gadus chalcogrammus*) and Anchoveta (*Engraulis ringens*) [1]. Given its affordability, significant nutritional content and exceptional gel properties, *K. pelamis* is an ideal raw material in surimi manufacturing [14].

Hence, the hypothesis was that surimi, consisting of *S. oualaniensis* and *K. pelamis* in specific proportions, may exhibit exceptional gel properties. To explore this potential, this investigation was conducted to assess the gel properties of mixed gels, and the underlying gelation mechanism was determined through molecular driving forces and the transformation of the secondary structure. Furthermore, the endothermal behavior of the myofibrillar protein was detected via differential scanning calorimetry. The objective of this study was to propose constructive strategies for enhancing gel characteristics and expanding the application potential of underutilized fish species in mixed surimi formulations.

## 2. Materials and Methods

### 2.1. Materials and Surimi Gel Preparation

The frozen *S. oualaniensis* and *K. pelamis* were purchased from China’s Yunfu Fishery Co., Ltd. in Wenchang city, Hainan Province, China. The fish were stored at −20 °C. All the chemicals used were of analytical quality and were procured from Xilong Scientific Co., Ltd. (Guangzhou, China).

The frozen *S. oualaniensis* and *K. pelamis* were thawed overnight at 4 °C. The carcass of *S. oualaniensis* was separated from other undesirable parts, leaving only the carcass, which was washed three times with ice water and then cut into pieces. *K. pelamis* is processed by extracting its internal organs, head, skin and bones. After the dark-colored muscle tissue was removed, the remaining flesh was sliced into pieces for later use. The processed *S. oualaniensis* and *K. pelamis* were separately blended in a meat grinder at low speed for 5 min each. The crushed *K. pelamis* was rinsed twice with ice water in a washing medium to a minimum ratio of 5:1 (*v*/*m*), followed by the addition of 0.3% sodium chloride solution. The washed substance was subsequently filtered with a 200-mesh gauze cloth. To prepare the gels, the moisture level of all the samples was adjusted to 80% by adding ice water [13]. The *K. pelamis* paste was mixed with *S. oualaniensis* paste at the following ratios, 1:0, 4:1, 3:2, 1:1, 2:3, 1:4 and 0:1 (*w*/*w*), and blended at low speed for another 10 min with the incorporation of 2.5% NaCl (*w*/*w*). The surimi obtained was subsequently inserted into a plastic casing measuring 3 cm in diameter, and the casing was tightly sealed at both ends. The surimi was then subjected to a water bath (HH-4, Changzhou Aohua Instrument Co., Ltd., Changzhou, China) at 40 °C for 1 h, followed by an additional poaching period of 30 min at 90 °C. After poaching, all the gel samples were immediately cooled in an ice bath and stored overnight at 4 °C.

### 2.2. Textural Profile Analysis

Textural profile analysis (TPA) of the samples was carried out on the basis of reference [16], with a minor adjustment. The samples were allowed to reach equilibrium at ambient temperature (28–30 °C) for a duration of 1 h prior to analysis. The test was conducted via a texture analyzer (TA. XT Plus, Stable Micro System, London, UK). The dimensions of the gel samples were 3 cm in diameter and 2.5 cm in height. The measurements were conducted via a P/36R columnar plunger, which was compressed at a strain level of 20% with a test speed of 1 mm/s. The application of force is directed along the vertical axis of the sample.

### 2.3. Penetration Test

The penetration test was executed by the texture analyzer mentioned above. A spherical probe (P/0.25S) was used. The speed of the indenter was adjusted to 1 mm/s, and the trigger force was set to 5 gf.

### 2.4. Water Holding Capacity and Cooking Loss Measurement

The water holding capacity (WHC) was determined according to the method proposed by reference [17] with a slight adjustment to ensure accuracy and reproducibility. The gel samples were sliced into small pieces with a uniform thickness of 1 cm, weighed *w*_1_, wrapped with double-layer filter paper and transferred into a 50 mL centrifuge tube. After centrifugation (TGL-16 M, Shanghai Luxiangyi centrifuge Instrument Co., Ltd., Shanghai, China) at 4000× *g* (gravitational acceleration) for 20 min at 4 °C, the gel sample was reweighed with *w*_2_. The WHC was calculated as follows:

(1) $$WHC\left(\%\right)=\frac{w_{2}}{w_{1}}\times 100\%$$

The measurement of cooking loss was conducted as described previously [18]. The filter paper was used to gently remove the moisture from the surface of the gel, and the mass was recorded before (*m*_1_) and after (*m*_2_) heating in a water bath with a retort pouch wrapped at 90 °C for 20 min. The cooking loss was quantified as follows:

(2) $$CL\left(\%\right)=\frac{m_{1}-m_{2}}{m_{1}}\times 100\%$$

### 2.5. Whiteness Measurement

L* (brightness level), a* (red–green index) and b* (yellow–blue index) were measured with a colorimeter (CS-820, CHNSpec, Hangzhou, China), and whiteness was calculated via the following formula [19]:

(3) $$Whiteness=100-{\left[{\left(100-L^{*}\;\right)}^{2}+a^{*2}+b^{*2}\right]}^{\frac{1}{2}}$$

### 2.6. Rheological Characteristics Measurement

#### 2.6.1. Apparent Viscosity

A rheometer (Discovery HR-2, TA, Cranston, RI, USA) was used to determine the apparent viscosity of the surimi [20]. Surimi paste samples were uniformly coated onto the test platform, with the flow sweep mode selected and a shear rate set at 0.1–500 s^−1^. The parallel plate geometry (60 mm) and a standard Peltier plate were employed, with the gap set at 1 mm. The viscosity-shear rate relationship was determined at a constant temperature of 25 °C.

#### 2.6.2. Dynamic Rheology

The storage modulus and loss modulus were measured with a rheometer operated in oscillating mode. The method is based on the reference [7] with slight modifications. Parallel plate geometries with a diameter of 60 mm and a gap of 0.45 mm were employed for the measurement. Two grams of surimi was placed onto the test platform with silicone oil covering the surface to create an air-isolated environment. The temperature range of the experiment was set between 25 °C and 90 °C, with a heating rate of 5 °C/min with 2% strain and a 0.1 Hz frequency.

### 2.7. Fourier Transform Infrared (FTIR) Spectroscopy

The gels were sliced to a thickness of 1.0 cm, and the absorption spectra of wavenumbers ranging from 4000 to 400 cm^−1^ were obtained via an IR spectrometer (Nicolet 50+ Continuum, Waltham, MA, USA). All the spectral data were analyzed via OMNIC V9.7 software. The calculation was performed to determine the proportion of secondary structure in proteins via PeakFit V4.12 software, according to reference [6].

### 2.8. Molecular Forces Measurement

The chemical forces of the protein in the surimi gel were determined on the basis of a previously reported method [21] with slight modifications. Surimi gel (1 g) was individually treated with five denaturing solutions (5 mL each), including SA (0.05 M NaCl), SB (0.6 M NaCl), SC (1.5 M urea + 0.6 M NaCl), SD (8 M urea + 0.6 M NaCl) and SE (0.5 M *β*-mercaptoethanol + 8 M urea + 0.6 M NaCl), followed by homogenization (8000 rpm, 1 min). These chemical bond disruptive solutions can degrade the primary molecular interactions within protein gels. Subsequently samples were incubated at 4 °C for 1 h to ensure a sufficient reaction, followed by centrifugation (5800× *g*, 20 min). A BCA protein assay kit (Beijing Labgic Technology Co., Ltd., Beijing, China) was used to measure the protein concentration in the supernatant. The protein concentrations in the supernatants of the five solutions are presented as C_SA_, C_SB_, C_SC_, C_SD_ and C_SE_ (g/L). The levels of ionic bonds, hydrogen bonds, hydrophobic interactions and disulfide bonds were determined on the basis of protein content and calculated as follows:

(4) $$Ionic\;bond=C_{SB}-C_{SA}$$

(5) $$Hydrogen\;bond=C_{SC}-C_{SB}$$

(6) $$Hydrophobic\;interaction=C_{SD}-C_{SC}$$

(7) $$Disulfide\;bond=C_{SE}-C_{SD}$$

### 2.9. Differential Scanning Calorimetry (DSC)

DSC measurements were conducted with a TA DSC250 (TA, Cranston, RI, USA). An aluminum pan was used to hermetically seal approximately 5 mg of sample. The samples were subsequently subjected to thermal analysis from 30 to 90 °C at a heating rate of 10 °C/min, with an empty pan serving as the reference. The positioning of the sample was meticulously executed to guarantee maximum surface contact with the underside of the aluminum pan.

### 2.10. Microstructure Observation

To observe the microstructure of the gel, the sample was sectioned into 3 mm × 3 mm × 3 mm cubes and freeze-dried. The dried sample was subsequently gold-coated and observed under a scanning electron microscope [20] with an acceleration voltage of 5 kV.

### 2.11. Data Analysis

The experimental data were determined by calculating the average and standard deviation (mean ± SD) of multiple measurements. Each replicate experiment was conducted with a minimum of three measurements. To assess the significance of treatment differences, one-way analysis of variance (ANOVA) was conducted via SPSS 26 software (IBM, Armonk, NY, USA). All visual images were created via Origin 2021 software (OriginLab Corporation, Northampton, MA, USA).

## 3. Results and Discussion

### 3.1. Gel Strength and WHC

**Figure 1:**
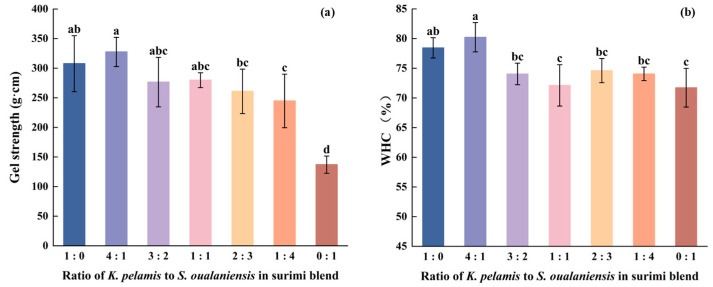
Gel strength (**a**) and WHC (**b**) of surimi gels. Dissimilar letters above the error bars represent significant differences among the samples (*p* < 0.05).

The gel strength plays a vital role in assessing the extent of cross-linking in a gel. The increased gel strength signifies superior gelation performance. As shown in Figure 1a, the addition of various proportions of *K. pelamis* significantly increased the gel strength of *S. oualaniensis* surimi (*p* < 0.05). The gel strength of the mixed surimi gel progressively increased with the increasing proportion of *K. pelamis*. When the addition of *K. pelamis* surimi reached a ratio of 4:1, the maximum gel strength was 327.48 g∙cm. Compared with *K. pelamis* alone (307.65 g∙cm) and *S. oualaniensis* alone (137.02 g∙cm), this probably suggests enhanced myosin protein-protein aggregation in the heat-induced gel. This result was similar to that of a previous study [13]. They reported the superior gel strength of a surimi blend consisting of croaker and short-bodied mackerel at a ratio of 1:2 to that of their individual counterparts.

During the heating process of salted surimi paste, protein molecules, especially myofibrillar proteins, unfold and expose reactive surfaces, thereby facilitating the formation of molecular bonds. When adequate bonding emerges, a 3D network is established, leading to ordered gel formation [22]. The active protease in *S. oualaniensis* contributed to the degradation of myofibrillar proteins, preventing protein-protein interactions and resulting in undesirable gel properties. According to a previous investigation [9], the addition of protease inhibitors effectively decreased the gel strength of squid, preventing the breakdown of MHC in squid surimi. According to reference [23], the presence of sarcoplasmic proteins, lipids and additional deposits could disrupt the gelling mechanism, thus leading to a diminished capacity for gelation.

Furthermore, the extent of protein-protein cross-linking is also responsible for the gelation ability. Transglutaminase (TGase), a water-soluble enzyme, is able to catalyze the cross-linking between lysine and glutamine, subsequently facilitating the aggregation of myofibrillar proteins at the setting temperature [24]. Reference [25] reported the impact of the presence of TGase on the thermal setting of surimi gels, revealing that TGase promoted MHC aggregation to form an ordered protein network. Reference [26] stated that the incorporation of salmon plasma protein in surimi may enhance the transglutaminase-mediated gelation process, resulting in an increase in gel strength. The activity of TGase varies significantly among different fish species [27]. The improved gel strength of pure *K. pelamis* could be attributed to its elevated endogenous TGase activity compared with that of *S. oualaniensis*. During heat treatment, proteins undergo conformational changes. The molecular interactions between proteins from diverse sources may facilitate alterations, providing favorable binding sites for enzymes. Thus, the mixture of surimi could strengthen protein-protein interactions, enhancing the extent of cross-linking, as evidenced by the DSC results.

The WHC is also a crucial index of gel quality, reflecting the capacity to retain water [28]. A high WHC indicates the formation of an ordered three-dimensional structure of surimi gel. The highest value of 80.23% was achieved in the mixed surimi at a ratio of 4:1 (K:S), whereas the lowest value of 71.72% was observed in the pure *S. oualaniensis* surimi, as shown in Figure 1b, in line with the trend of gel strength. A sufficient addition of *K. pelamis* muscle enhanced protein-water interactions, thereby decreasing water fluidity. Similarly, soy isolate protein has been demonstrated to enhance interactions with water molecules, thereby increasing its capacity to anchor these molecules [29]. According to the findings reported in reference [30], surimi samples exhibiting more intact myosin heavy chains demonstrated superior performance in expressible moisture content tests. The unfavorable WHC may be attributed to the denaturation of myofibrillar molecules, which reduces water retention within the surimi gel network [31]. The active proteolysis in *S. oualaniensis* disrupted the native conformation of myosin proteins, thereby influencing their water-holding capacity, suggesting that the WHC was directly linked to the structural integrity of myofibrillar molecules.

### 3.2. Textural Properties

Textural profile analysis can imitate the oral actions of the tongue and teeth to reflect the structural characteristics of gels. The TPA results, including hardness, springiness, cohesiveness, gumminess, chewiness and resilience, are shown in Table 1. The hardness, gumminess and chewiness values tended to increase with increasing proportions of *K. pelamis* in the surimi blend. Compared with those of the pure *S. oualaniensis* surimi gel (0:1), these three parameters significantly increased in the mixed gels (*p* < 0.05). These results demonstrated that the addition of *K. pelamis* to the surimi blend promoted the aggregation of rigid protein, contributing to the enhancement of gel quality. This outcome is aligned with the study conducted in reference [14]. According to previous reports [32,33], the addition of exogenous animal proteins could facilitate the formation of a more compact gel matrix, thus improving the hardness and gumminess of surimi gels. Similarly, the combination of *S. oualaniensis* and *K. pelamis* resulted in a denser structure, leading to increased hardness and gumminess values. There were no significant differences in the springiness or cohesiveness values of the gel among all the samples, irrespective of the mixture ratio (*p* > 0.05). Thus, mixed surimi with an appropriate hardness and chewiness could be preferable for consumers. The resilience, which represents the capacity to swiftly recover from deformation under pressure, showed a peak in the pure *S. oualaniensis* gel. Paramyosin, a significant ingredient of the myofibrillar fraction only in invertebrates, can affect actomyosin gel viscoelasticity [34,35]. The marked differences in resilience among these samples may be attributed to the special components within the myofibrillar proteins of *S. oualaniensis*.

### 3.3. Cooking Loss

**Figure 2:**
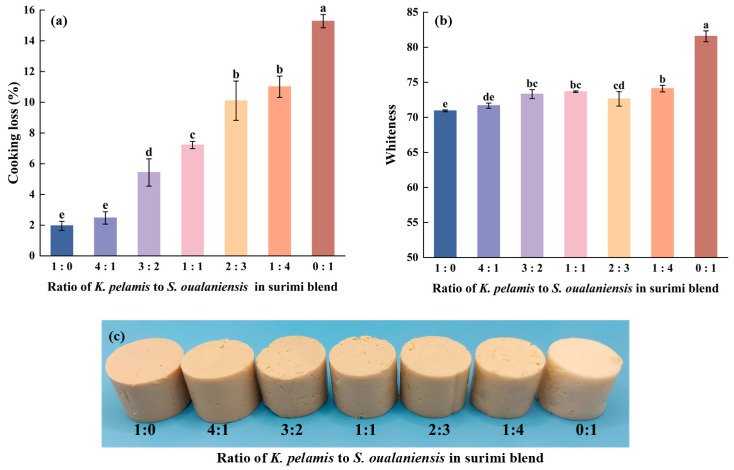
Cooking loss (**a**), whiteness (**b**) and appearance (**c**) of surimi gels. Dissimilar letters above the error bars represent significant differences among the samples (*p* < 0.05).

During the process of cooking, the water trapped within the network structure is lost because of the denaturation of protein and collagen contraction caused by the high temperature. The cooking loss (CL) data are shown in Figure 2a. The pure *S. oualaniensis* surimi gels presented a peak CL value of 15.28%, whereas the addition of *K. pelamis* muscle significantly reduced the CL value (*p* < 0.05). When the *K. pelamis* to *S. oualaniensis* ratio was 1:4, the CL value decreased to 11.01%, representing a reduction of 28% compared with that of the pure *S. oualaniensis* surimi gel. When the ratio reached 4:1 (K:S), the cooking loss was 2.47%, which was not significantly different from that of pure *K. pelamis* surimi gel. Reference [36] reported that the high cooking loss of giant squid might be attributed to the rapid aggregation of its myofibrillar proteins during gelation, which facilitates the rapid expulsion of water from the surimi, leading to considerable water loss and the development of an irregular gel structure. The decrease in the value of CL was evidently correlated with the progressive expansion of *K. pelamis*, indicating the formation of a more ordered surimi gel structure, as shown in the results of scanning electron microscopy. In addition, the change in CL may be related to the exudation of water-soluble substances with the degradation of network-like structures while heating [37].

### 3.4. Color

Whiteness serves as another significant index for assessing the quality of surimi products. The whiteness testing results are shown in Figure 2b. The peak value of 81.57 was observed in the individual *S. oualaniensis* group. Nevertheless, the addition of *K. pelamis* had a significant effect on the whiteness of the surimi gels (*p* < 0.05). With increasing proportions of *K. pelamis* in the mixture, the whiteness decreased from 74.11 to 70.94. This phenomenon could be attributed to the greater extent of lipid oxidation in *K. pelamis*, facilitating the formation of aldehydes or carbonyl compounds. These compounds then interact with amino groups in proteins via Maillard reactions, which produce colored reaction products that reduce whiteness [38]. Compared with pure *K. pelamis* surimi gel, mixed surimi gels exhibiting greater whiteness may be more appealing to consumers [39]. The denser network structure may also lead to a reduction in light transmission through the gel, thus contributing to the observed decrease in whiteness [40]. Reference [41] reported that the whiteness of blended gels composed of silver carp and scallops varied from 73.51 to 79.02 depending on the different ratios of the components. The slight difference in whiteness between the two types of blended surimi is likely attributable to variations in the raw materials.

### 3.5. Rheological Properties

#### 3.5.1. Dynamic Rheological Properties

**Figure 3:**
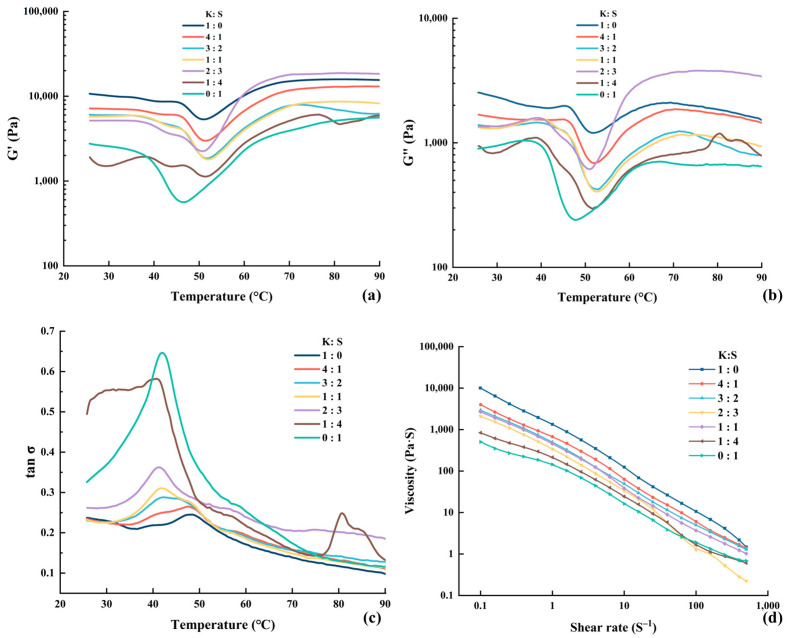
Modifications in the storage modulus (**a**), loss modulus (**b**), loss tangent (**c**) and apparent viscosity (**d**) of surimi. K, *K. pelamis*; S, *S. oualaniensis*.

The values of the storage modulus (G′), loss modulus (G″) and dissipation tangent are presented in Figure 3. G′ is the energy stored with elastic deformation, indicating the elastic property and strength of surimi. The variation trends of G′ were basically consistent among all the samples. As shown, the value of G′ showed negligible or marginal fluctuations before the temperature reached 37 °C. The G′ subsequently tended to decrease with increasing temperature, which may be attributed to the occurrence of proteolytic activity. Reference [42] reported that subjecting raw materials to 55 °C could result in the activation of endogenous proteases, leading to protein denaturation and degradation of the network structure. The hydrophobic groups of the proteins were exposed due to disintegration of the gel matrix, increasing water mobility and escape, thereby facilitating modori and consequently decreasing G′. The peaks were subsequently observed at approximately 52 °C, with the exception of pure *S. oualaniensis*, which exhibited a peak at 45 °C. According to reference [43], chicken plasma protein (CPP) functions as an enzyme inhibitor in surimi gels, effectively preventing the degradation of MHC and consequently promoting the formation of ordered fibrillar structures. The higher peak temperature observed likely suggests that the inclusion of *K. pelamis* suppressed protease activity in *S. oualaniensis* muscle, generating a lower rate of proteolysis. After reaching the lowest G′, the myosin heavy chain and actin denature, leading to the exposure of hydrophobic groups and -SH, which promotes the formation of an organized cross-linked structure through chemical forces. As a result, a steady increase in G′ was observed from 52 °C to 70 °C [17]. From 70 °C to 90 °C, G′ remained relatively stable or exhibited a slight decrease. This probably suggested that the gel structure was nearing its stable conformation. After the denaturation and structuring reactions are complete, increased temperature raises kinetic energy and molecular vibration, thereby reducing G′ due to kinetic effects.

Notably, *K. pelamis* surimi presented a decrease peak at approximately 42 °C, which gradually diminished with the addition of *S. oualaniensis* and eventually vanished. This phenomenon demonstrated that other protein deposits might exist in *K. pelamis* paste, which warrants further investigation. According to reference [44], a high value of G′ represents the appearance of a tight protein structure as a result of the creation of *ε*-(γ-glutamyl) lysine bonds induced by endogenous TGase. As shown in Figure 3a, the value of G′ increased with increasing proportion of *K. pelamis* in the surimi blend, leading to improved cross-linking between proteins, which was supported by increased molecular driving forces (as shown in the results of Molecular Forces) and a greater share of β-sheets (as shown in the results of FTIR). In addition, an extraordinary increase in the G′ value was observed at a ratio of 2:3 (K:S) from approximately 50 °C to 70 °C. The air-isolated environment created by silicone oil may have been destroyed during testing, causing the oxidation of lipids in surimi. The free radicals generated during the lipid oxidation process can induce protein oxidation, leading to the transformation of sulfhydryl groups into disulfide linkages [45]. Consequently, the extent of protein cross-linking was increased, thereby contributing to the pronounced increase in G′.

G″ denotes the dissipation and energy loss during oscillation, representing the viscous behavior of surimi. It is evident that G″ < G′ throughout the entire process, indicating a higher elasticity and lower viscosity of surimi. Thus, the surimi exhibited a predominantly elastic texture during thermal processing, which was consistent with reference [46]. The trends of G″ were similar to those of G′ within the temperature range from 25 °C to 70 °C. However, the G″ value gradually decreased when the temperature exceeded 70 °C, indicating the persistence of residual viscosity within the heat-induced gels [47]. The loss tangent (tan δ) is presented in Figure 3c. The value of tan δ was consistently less than 1, indicating that the surimi was a gel that was mainly elastic.

#### 3.5.2. Apparent Viscosity Profile

The rheological properties of fluids are partially embodied in the viscosity of materials, manifesting the velocity and resistance exhibited by protein solutions when subjected to external forces. The apparent viscosity profile is shown in Figure 3d. With an increasing shear rate, the viscosity initially rapidly decreased, followed by a subsequent plateau. As the shear rate increased, the entanglement points in the fish mince system were untangled and aligned in an orderly manner along the direction of shear flow, resulting in a decrease in viscosity until equilibrium was reached. The phenomenon wherein the apparent viscosity of a sample decreases with increasing shear rate is referred to as the shear-thinning effect, indicating a flow behavior index below 1 [48]. The shear-thinning behavior suggested that surimi can be classified as a pseudoplastic fluid [49]. The low viscosity of the *S. oualaniensis* paste may be related to the high degree of proteolysis induced by its internal proteinase, which enhanced the conformational extension of the protein. The transformation of protein accelerated the molecular orientation, thus decreasing the flow resistance.

### 3.6. Molecular Forces

The development of the network structure and gel properties in surimi gels is driven primarily by hydrogen bonds, ionic bonds, disulfide bonds and hydrophobic interactions. Specific chemical substances in solution can effectively breakdown the respective chemical linkages, thereby increasing the solubility of proteins. Consequently, the concentration of soluble proteins in the solution serves as a quantifiable indicator of the relative strength of various chemical interactions.

**Figure 4:**
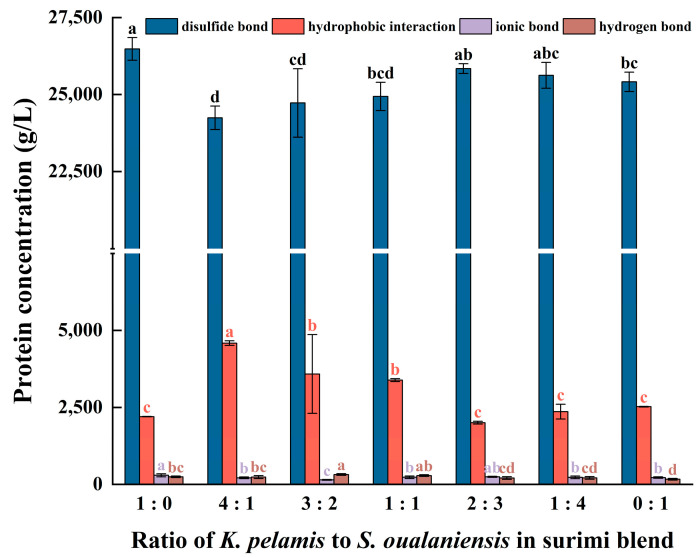
The content of molecular forces in surimi gels includes disulfide bonds, hydrophobic interactions, ionic bonds and hydrogen bonds. Dissimilar letters above the error bars represent significant differences among the samples (*p* < 0.05).

Thermal unfolding, the initial step of protein denaturation, induces the exposure of inner hydrophobic cores and sulfhydryl groups [50,51]. To maintain the thermostability and decrease the entropy value of the system, adjacent proteins react via protein-protein hydrophobic interactions and disulfide bonds, subsequently facilitating the aggregation of proteins and the formation of network gels [52]. As shown in Figure 4, the content of disulfide bonds and hydrophobic interactions significantly surpassed that of ionic bonds and hydrogen bonds. This finding suggests that the stability of a protein’s three-dimensional structure mostly depends on disulfide bonds and hydrophobic interactions, which aligns with the findings reported in the literature [53]. The gel of *K. pelamis* presented the highest content of disulfide bonds, whereas the surimi mixture (K:S = 4:1) presented the highest level of hydrophobic interactions. The hydrophobic interactions increased with increasing *K. pelamis* content in the surimi blend, and the content of disulfide bonds slightly decreased. The protein molecules from the two fish species exhibited intermolecular interactions during the process of thermal unfolding, and they gradually aggregated into granules through hydrophobic interactions. The formation of rigid protein aggregates reduced protein chain flexibility, restraining the reaction of neighboring sulfhydryl groups and thus decreasing the content of disulfide bonds in mixed surimi. This modification of molecular forces could be pivotal in enhancing the suboptimal texture properties of *S. oualaniensis* gels. Moreover, the trend of the hydrophobic interactions aligns with those observed for the WHC and gel strength mentioned above, suggesting that the hydrophobic interactions had a more significant effect on the gel properties.

### 3.7. DSC

**Figure 5:**
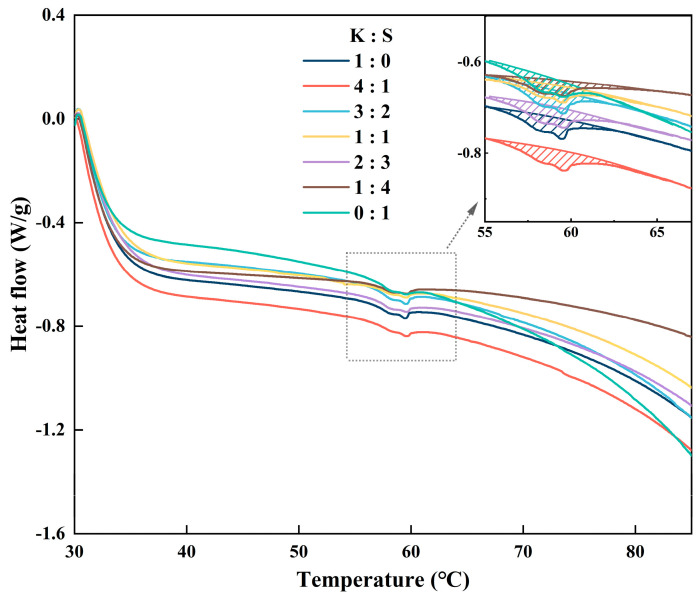
DSC thermograms of surimi samples. K, *K. pelamis*; S, *S. oualaniensis*.

The denaturation of proteins is caused by sufficient heat input, which disrupts the stability of chemical bonds that sustain the network structure. Here, differential scanning calorimetry was applied to reveal the endothermic behavior of surimi protein. The heat of the endothermic peak is considered the enthalpy in the process of protein denaturation (ΔH), and the temperature of the peak maximum (T_p_) is the transition temperature of the protein [54]. Myofibrillar protein is the predominant component of surimi products. The typical thermogram of surimi products exhibits two distinct peaks, with the initial peak corresponding to the heat-induced transition of myosin and the subsequent peak representing that of actin [55]. The thermogram of the surimi blend is shown in Figure 5. Notably, only the transition of myosin was detected when the experimental temperature of the salted surimi products was varied. This phenomenon was also observed in the work of reference [56], which demonstrated that the addition of salt to surimi may lead to a higher actin denaturation temperature.

The endothermic peak of myosin occurred at temperatures ranging from approximately 55 °C to 65 °C, and a slight difference in denaturation temperature emerged. The transition temperature (T_p_) and thermal enthalpy (ΔH) were documented in Table 2. Compared with the values of 0.1149 J/g (0:1) and 0.1304 J/g (1:0) for the control samples, the mixture significantly improved the value of endothermic enthalpy, indicating the formation of superior cross-linking of myosin. Reference [57] reported that T_p_ serves as an indicator of the disturbance of protein aggregation caused by hydrophobic interactions and hydrogen bonds. The maximum T_p_ was observed at a ratio of 4:1 (K:S), demonstrating strengthened intermolecular and intramolecular interactions, which was consistent with the results recorded in Figure 4.

### 3.8. FTIR

**Figure 6:**
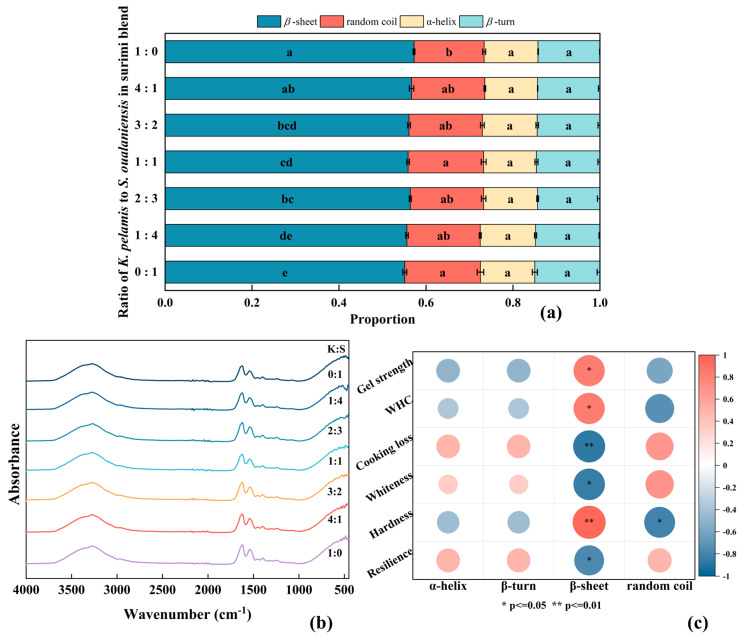
Secondary structure (**a**), FTIR spectrum (**b**) and correlation analysis (**c**) of surimi gel. K, *K. pelamis*; S, *S. oualaniensis*. Dissimilar letters above the error bars represent significant differences among the samples (*p* < 0.05).

FTIR spectroscopy is frequently utilized for the effective analysis of protein secondary structure in scientific research. The amide I peak corresponds to the stretching vibration of the C=O bond within the range of 1600–1700 cm^−1^, which is used to determine the proportion of the secondary structure. The spectral ranges from 1600 to 1640 cm^−1^, 1640 to 1650 cm^−1^, 1650 to 1660 cm^−1^ and 1660 to 1700 cm^−1^ were attributed to *β*-sheets, random coils, α-helices and *β*-turn structures, respectively [46]. The spectrum and secondary structure content are documented in Figure 6. According to Figure 6a, the *β*-sheet is the dominant structure in surimi. Relevant studies have demonstrated that random coils and *β*-turns are categorized as disordered structures, whereas *β*-sheets and α-helices are classified as organized structures [58]. No statistically significant differences were detected in the proportions of α-helices or β-turns among all the samples (*p* > 0.05). The addition of *K. pelamis* surimi significantly increased the proportion of *β*-sheets and reduced random coil conformation in the gel (*p* < 0.05), indicating that the internal structure underwent a transition from disorder to order. Furthermore, the trend of *β*-sheets resembled that of hydrophobic interactions in the surimi blend. The alteration of the secondary conformation might promote the exposure of internal hydrophobic regions, thereby facilitating myosin aggregation and leading to the formation of compact protein aggregates.

To further investigate the relationship between the secondary structure and gel properties, correlation analysis was conducted, and the corresponding results are shown in Figure 6c. The hardness was positively correlated with the proportion of *β*-sheets (*p <* 0.01), indicating the formation of an enhanced network structure, which is consistent with previous studies [47,59].

### 3.9. Microstructure

**Figure 7:**
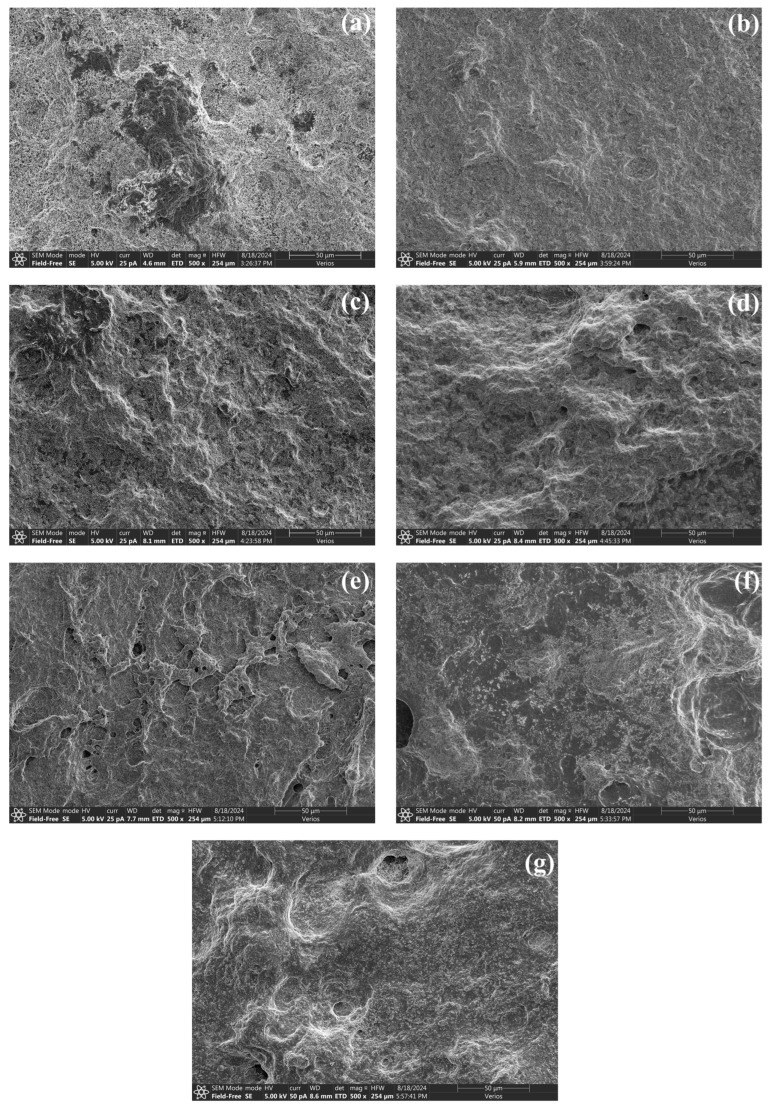
Microstructure of surimi gels. The letters (**a**–**g**) represent the blend surimi at ratios of 1:0, 4:1, 3:2, 1:1, 2:3, 1:4 and 0:1 (K:S), respectively. K, *K. pelamis*; S, *S. oualaniensis*.

In this study, the three-dimensional microstructure of heat-induced surimi gels was revealed by scanning electron microscopy (Figure 7). Significant differences between pure *S. oualaniensis* and the other samples were observed. The pure *S. oualaniensis* protein gel presented a relatively uneven surface topography with numerous prominent pores, which is consistent with its loose reticular structure. The incorporation of *K. pelamis* led to a gradual increase in the gel surface area of the surimi mixture, characterized by improved smoothness and reduced pore size. In particular, the surimi exhibited a well-filled appearance at a ratio of 4:1 (K:S), with a uniform surface and compact structure, demonstrating that high hydrophobic interactions contributed to the creation of protein aggregates with smooth surfaces. The variation in microstructure shown above was similar to the trend in gel strength. The results also supported the decreased water mobility, as highlighted in Figure 1. Furthermore, the presence of shrunken holes indicates that *K. pelamis* paste might function as a filler to enhance the structure of the gels.

## 4. Conclusions

In summary, we characterized the gel properties and gelation mechanism of surimi blends consisting of *S. oualaniensis* and *K. pelamis* at various ratios. According to the gel property results, a ratio of 4:1 (K:S) was the optimal ratio for the surimi mixture, which resulted in superior gel strength and WHC. The increase in *K. pelamis* in the blend led to an improvement in the hardness, chewiness and cooking loss of the gels, which was harmful to their resilience and whiteness. FTIR revealed that the addition of *K. pelamis* resulted in a secondary structural transformation of random coils to *β*-sheets, facilitating the formation of an ordered network and thus increasing the values of G′ and G″. The data of the molecular forces demonstrated that the disulfide linkages and hydrophobic interactions were the predominant driving forces in maintaining the stable structure of the gel, while the latter was identified as the key factor influencing the performance changes in the mixed gels. The increasing number of hydrophobic interactions promoted the aggregation of myofibrillar proteins, leading to the emergence of a denser three-dimensional structure. DSC analysis revealed a relatively high transition temperature and increased endothermal empathy in the surimi mixture, suggesting an increased degree of cross-linking. SEM revealed a smoother surface and shrunken cavities in the surimi blend in the presence of *K. pelamis*, which could help to enhance the sensory quality of the product by improving its appearance and texture to meet consumer demands. This work provides in-depth insights into the gelation mechanism of mixed surimi. Compared with single surimi, mixed surimi exhibited superior mechanical properties and enhanced thermal stability, thereby improving its reliability for both industrial production and home cooking applications. This enhancement facilitates the broader utilization of underutilized fish resources and paves the way for expanding the application of underutilized fish species from the South China Sea.

## Figures and Tables

**Table 1:** TPA results for the surimi blend.

K:S	Hardness (gf)	Springiness (Ratio)	Cohesiveness (Ratio)	Gumminess (gf)	Chewiness (gf)	Resilience (Ratio)
1:0	2410 ± 286 ^a^	0.96 ± 0.02 ^ab^	0.87 ± 0.06 ^a^	2083 ± 238 ^a^	2003 ± 236 ^a^	0.50 ± 0.05 ^b^
4:1	1912 ± 150 ^b^	0.96 ± 0.01 ^ab^	0.86 ± 0.06 ^a^	1643 ± 108 ^b^	1574 ± 115 ^b^	0.49 ± 0.05 ^b^
3:2	1434 ± 75 ^c^	0.97 ± 0.02 ^ab^	0.87 ± 0.06 ^a^	1246 ± 74 ^c^	1207 ± 93 ^c^	0.50 ± 0.05 ^b^
1:1	1416 ± 114 ^c^	0.96 ± 0.03 ^b^	0.87 ± 0.05 ^a^	1228 ± 112 ^c^	1175 ± 128 ^c^	0.50 ± 0.04 ^b^
2:3	1328 ± 158 ^cd^	0.97 ± 0.02 ^ab^	0.88 ± 0.05 ^a^	1164 ± 150 ^cd^	1128 ± 160 ^cd^	0.52 ± 0.04 ^ab^
1:4	1179 ± 55 ^d^	0.96 ± 0.02 ^ab^	0.88 ± 0.05 ^a^	1038 ± 84 ^d^	1002 ± 98 ^d^	0.52 ± 0.04 ^ab^
0:1	941 ± 40 ^e^	0.98 ± 0.02 ^a^	0.89 ± 0.05 ^a^	835 ± 45 ^e^	818 ± 54 ^e^	0.56 ± 0.04 ^a^

K, *K. pelamis* surimi; S, *S. oualaniensis* surimi. Different letters under the same parameter represent significant differences among the samples (*p* < 0.05).

**Table 2:** The transition temperature (T_p_) and transition enthalpy (ΔH) of the surimi samples.

K:S	T_p_ (°C)	ΔH (J/g)
1:0	59.61	0.1304
4:1	59.70	0.1542
3:2	59.61	0.1321
1:1	59.56	0.1303
2:3	59.51	0.1452
1:4	59.56	0.1270
0:1	59.45	0.1149

K, *K. pelamis*; S, *S. oualaniensis*.

## Data Availability

The original contributions presented in the study are included in the article, further inquiries can be directed to the corresponding author.

## Abbreviations

The following abbreviations are used in this manuscript:

DSC	Differential scanning calorimetry
